# Acceptability of donor breastmilk banking among health workers: a cross-sectional survey in Zimbabwean urban settings

**DOI:** 10.1186/s13006-020-00283-y

**Published:** 2020-05-11

**Authors:** Dexter T. Chagwena, Felistas Mugariri, Bhekimpilo Sithole, Steady Faith Mataga, Ruvimbo Danda, Tonderayi M. Matsungo, Charles C. Maponga

**Affiliations:** 1 School of Pharmacy, College of Health Sciences, University of Zimbabwe, Harare, Zimbabwe; 2 Nutri@ctive Zimbabwe, 96 Golden Stairs Rd, Mt Pleasant, Harare, Zimbabwe; 3 Institute of Food, Nutrition and Family Sciences, Mt Pleasant, Harare, Zimbabwe; 4 Ministry of Health and Child Care, Causeway, Harare, Zimbabwe; 5 UNICEF, 6 Fairbridge Avenue, Belgravia, Harare, Zimbabwe

**Keywords:** Breastmilk banks, Knowledge, Health workers, Breastfeeding, Urban setting, Zimbabwe

## Abstract

**Background:**

The World Health Organization (WHO) recommends that donor human milk is superior to artificial infant formula in situations where the baby cannot feed on the mother’s breastmilk**.** The purpose of this study was to determine the acceptability of donor human milk banking among health workers in Zimbabwean urban settings.

**Methods:**

A cross sectional study was conducted among 535 health workers and 15 key informants. Three referral hospitals were purposively selected and systematic random sampling was used to select the health workers. The study was conducted between October 2017 and October 2018.

**Results:**

The concept of donor human milk banking was acceptable among health workers. One-third (31%) of the study participants reported that they would accept donor breastmilk for their children while 56% of them would encourage their clients to donate breastmilk. Acceptance of donor human milk banking was associated with a high level of knowledge on breastmilk banks (*p* = 0.009) and the study participants’ health profession (*p* = 0.001). Clinical staff were more receptive to donor human milk banking compared to non-clinical health workers. Donor human milk banking was not associated with religion (*p* = 0.498) or marital status (*p* = 0.714).

**Conclusions:**

The results showed that health workers and policy informers would accept the establishment of breastmilk banks subject to resource availability. Commitment to the establishment of breastmilk banks was moderately acceptable among opinion leaders responsible for spearheading health and nutrition policies.

## Background

The WHO recommendation that donor breastmilk is the next best option when mother’s own milk is unavailable has highlighted the concept of donor breastmilk banking in developing countries [1–4]. However, successful implementation of donor human milk (DHM) banks in settings with high HIV prevalence has been questionable especially when compounded by cultural issues. In countries where DHM banks have been successfully established the crucial role played by health workers to ensure acceptability and success of DHM banks has been stressed [5, 6].

Breastfeeding benefits both the child and the mother resulting in improved future intelligence, economic development and reduced mortality [7–12]. Breastmilk, whether mother’s own milk or DHM, is viewed as superior to infant formula even in HIV prevalent areas [1, 13, 14]. In instances where breastfeeding is not possible, either breastmilk substitutes or donor breastmilk is required. WHO recommends the use of infant feeding options that are culturally acceptable to ensure optimal health for the child [15]. Zimbabwe reports high breastfeeding rates, with 98.1% of children breastfed at some point before reaching 24 months [16]. Exclusive breastfeeding (EBF) is currently estimated at 61% and efforts to encourage EBF are mainly advanced through the Baby-Friendly Hospital Initiative (BFHI) and community-based infant and young child feeding (IYCF) promotion led by the Ministry of Health and Child Care [17, 18].

The country follows the universal WHO recommendations for all infants despite HIV status of the mother of exclusive breastfeeding for 6 months, followed by the introduction of nutritious adequate complementary foods and continued breastfeeding for 24 months or beyond [13, 19, 20]. Where breastfeeding is not possible, infant formula is usually the alternative infant-feeding option in spite of the campaign for EBF within the initial 6 months of life [18]. Common cases of low birth weight, premature births, and orphans in the country’s hospitals have motivated the government to explore the promotion of breastmilk feeding utilizing donor breastmilk banks [21]. Prevalence of low birth weight is currently estimated at 10%, while cases of preterm babies are common in tertiary hospitals although scientifically reported national data are scarce [16]. Evidence also indicates that maternal deaths are high during the peak childbearing age, suggesting high numbers of orphans under 2 months of age [16]. In line with the country’s policy to ensure all children receive breastmilk, DHM banks provide an opportunity to ensure universal access to breastmilk for all children aged 0–24 months. Clearer understanding of the perceptions of health workers and policy makers could facilitate more effective interventions.

Breastmilk protects preterm babies against necrotizing enterocolitis, sepsis, improves neurodevelopmental outcomes, tolerance of feedings and shortens the length of stay in neonatal intensive care units (NICU) providing direct cost savings [3, 22–27]. DHM is associated with increased EBF rates, and increased awareness of families and NICU staff on the value of breastfeeding. Further, DHM banking provides an opportunity for lactation support to mothers as well as protection of breastfeeding through an integrated framework of newborn care [28–30].

Developing countries have demonstrated capacity to manage breastmilk banks successfully. Brazil has the largest network of DHM banks in the world with over 219 banks. Other African countries such as South Africa and Kenya have established a network of DHM banks despite facing epidemics of infectious diseases such as HIV, TB and hepatitis [5, 6, 31–34]. The role of health workers has been crucial in the success of DHM banks. However, health workers’ attitudes, fragmented systems, lack of government policy support, maternal and community negative perceptions and detrimental cultural practices often prevent success of DHM banks [29, 33, 35]. Health workers play a key role in motivating and influencing mothers to donate breastmilk. In the Brazilian experience, mothers obtained information about a DHM bank during their stay at a hospital and were influenced to donate breastmilk by a health worker [5]. This strategy indicated that success or failure of DHM banks is hinged on health workers.

In Zimbabwe, limited data exists on how health workers perceive the practice of DHM banks. This study was designed to assess the acceptability and perceptions of DHM banks among health workers in a low-income urban setting in the context of high HIV prevalence.

## Methods

### Study setting

The study was conducted at Parirenyatwa Group of Hospitals, Harare Central Hospital and Mpilo Central Hospital in the two major cities of Zimbabwe between October 2017 and October 2018. These are the major referral hospitals in the country. These hospitals are potential pilot locations for establishing breastmilk banks in the country. Harare is the capital city of Zimbabwe and has a total population of 2,123,132 (according to the 2012 Census) with a proportion of women of child bearing age of 58.6%, and children below the age of five at 14.1%. Bulawayo is the second largest city in the country [36].

### Study design

A cross-sectional survey was conducted among health workers at three tertiary health institutions. A mixed methods approach was applied using both quantitative and qualitative methods. Key informant interviews were conducted with hospital management to explore the readiness of Zimbabwe tertiary hospitals to implement donor breastmilk banks, in addition to opinion leaders involved in health and nutrition policies.

### Study population, sampling and eligibility criteria

The three referral hospitals were purposively selected, and a systematic random sampling method used to select health workers to be interviewed from maternal, medical and paediatric wards as well as nutrition departments. Sample size was based on Naing’s formula using α = 0.05 and 95% significance level and 50% estimated prevalence of knowledge, attitude and perceptions [37]. A sample size of 384 health workers from the three hospitals was found to be adequate. Fifteen key informants were purposively selected so as to include all relevant administrative departments at each health institution and national management in the Ministry responsible for Health and Child Care.

Health workers working at Parirenyatwa Group of Hospitals, Harare Central Hospital and Mpilo Central Hospital at the time of data collection were eligible to participate in the study. Health workers were grouped based on their professional roles; doctors, nurses, midwives, nutrition and general staff. Management staff of the tertiary hospitals including head of departments from both clinical and administrative departments were included in the study as key informants. Opinion leaders in the health and nutrition sector including key development partners in these sectors were included as key informants as well.

### Interviewing and data collection

**Fig. 1:**
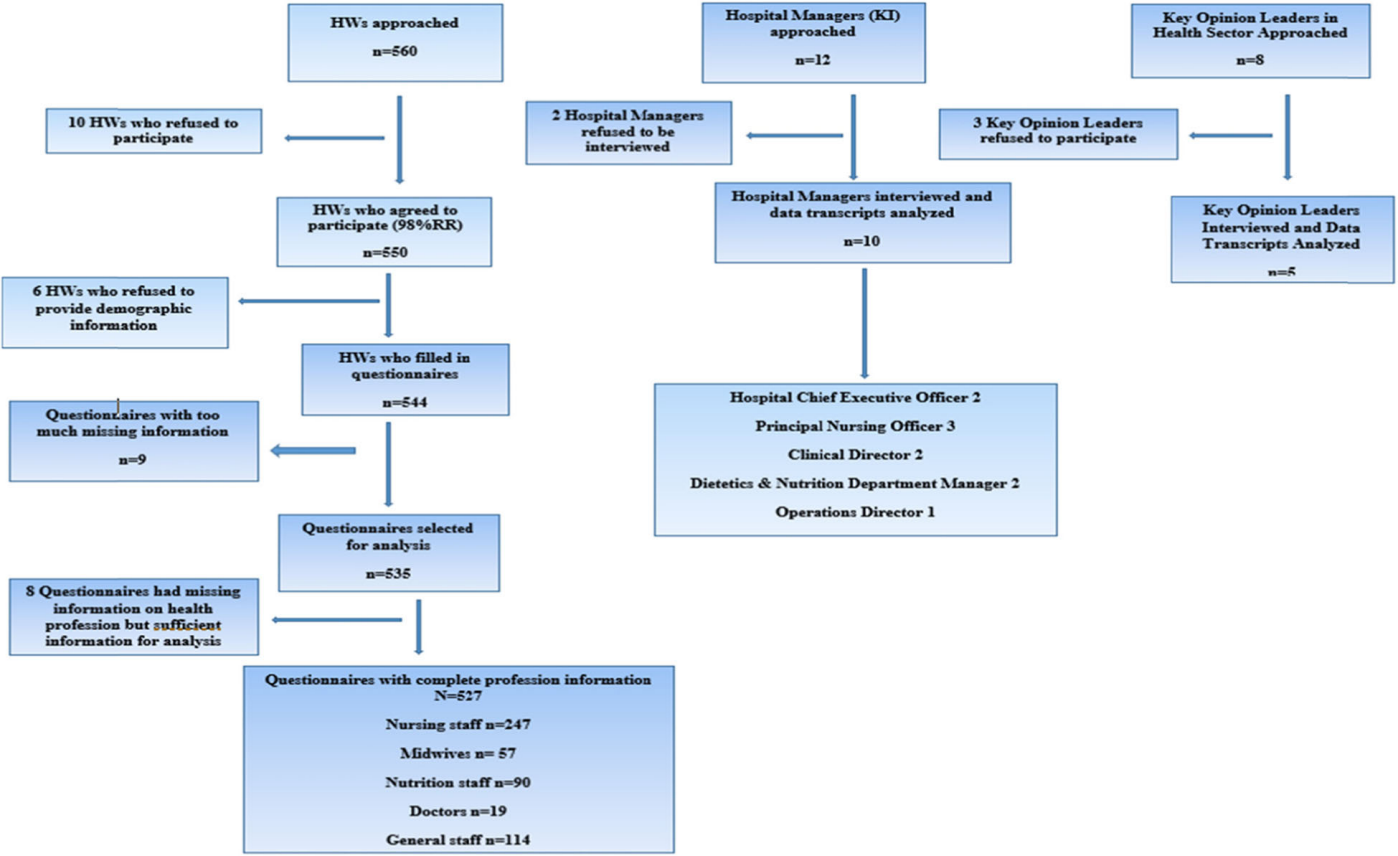
Flow chart of study participants selection

Health workers were interviewed using a structured, self-administered questionnaire. The questionnaire was pretested among health workers at one polyclinic in Harare. Key informant interviews (KIIs) were conducted with hospital management, the Ministry of Health and Child Care’s opinion leaders. Hospital managers were selected from the same tertiary hospitals as health workers and interviewed separately. The Hospital Managers interviewed included two Hospital Chief Executive Officers, three Principal Nursing Officers, two Clinical Directors, two Dietetics and Nutrition Department Managers and one Operations Director. The Opinion Leaders interviewed included two Family Health and Nutrition Directors, two Nutrition Technical Specialists and the President of the Zimbabwe Midwives Association (see Fig. 1).

### Statistics and data analysis

The data were analyzed to determine suitability, reliability, adequacy and accuracy using STATA V13 software package (StataCorp, 2013) [38]. Unless otherwise stated, all analyses of the quantitative data were presented as mean ± SD and the level of significance was set at *P* < 0.05. Chi-squared testing was used to test for significant difference in characteristics related to knowledge and acceptance of BM banks. Binary logistic regression analysis was employed to test for association between health workers’ personal and professional characteristics with their acceptance of BM banks. Qualitative data were analyzed using content analysis of themes employing Microsoft Excel. All interviews were transcribed, and a cross-case analysis was used to identify themes from the combined responses of all key informants.

The data were initially examined by the themes and topics employed in the interview guide to establish participants’ knowledge and perceptions on establishing breastmilk banks in the country. An inductive analysis was then used to identify further themes, sub-themes and patterns in the data. The following steps were employed for the qualitative data analysis. 1) Reading and re-reading the responses to become familiar with the text and begin developing codes. 2) Coding the data to identify themes and sub-themes. 3) Displaying details of categories and themes (e.g. identifying variations within each theme, noting differences between individuals and among sub-groups, and exploring nuances in the text). 4) Reducing the data to essential points, and 5) developing an overall interpretation based on this process [39]. Key themes were generated from full transcripts of interviews from key informants and opinion leaders. This was conducted by employing content analysis generating themes and sub-themes. Data triangulation was achieved using qualitative data from KIIs to corroborate the information reported by health workers from the structured questionnaires. The primary data analysis was conducted by DTC and FM. To avoid bias and ensure consensus, data checking and review of the content analysis was conducted by a senior author (TM).

## Results

The survey results were presented and compared with verbal quotes from the key informant interviews. Of the 535 health workers who participated in the study 72% were female, 47% were nurses, 66% were married and 5% had previously worked in a setting with a breastmilk bank (Table 1).

**Table 1:** Socio-demographic characteristics of Health Workers in Zimbabwean Tertiary Hospitals (*n* = 535)

Characteristics	n	%
*Tertiary Hospitals (n = 535)*		
Parirenyatwa Hospital	237	44
Harare Hospital	220	41
Mpilo Hospital	78	15
*Gender of Respondents (n = 535)*		
Male	150	28
Female	385	71
*Profession of respondents (n = 527)*		
Nursing	247	47
Midwife	57	11
Nutrition	90	17
Doctor	19	4
General staff	114	22
*Marital status of respondents (n = 528)*		
Single	146	28
Married	348	66
Divorced	9	2
Widow	25	5
*Respondents with children (n = 535)*	426	80
*Religion (n = 535)*		
None	7	1.31
Christianity	517	96.64
Muslim	3	0.56
African traditional religion	3	0.56
Apostolic sector	5	0.93

*Where n < 535 represents participants with missing data on those variables

### Knowledge on human milk banking and infant feeding

More than half the health workers (58%) were aware of breastmilk banks, and more than two-thirds (71%) knew of the human milk banking practice. Personal knowledge, the workplace and internet were reported as the major source of information regarding breastmilk banks by health workers (Table 2).

**Table 2:** Awareness and common sources of information regarding breastmilk banks

	n	%
*Awareness of BM Banks (n = 535)*	306	58
*Correct definition of breastmilk bank (n = 525)*	371	71
*Health previously exposed to donor breastmilk banks (n = 535)*	27	5
*Common sources of information on breastmilk milk donation (n = 365)*		
Formal education	38	10
Internet	56	15
Media	11	3
Other people	48	13
Workplace	93	25
Personal knowledge	119	33

*Where n < 535 represents participants with missing data on those variables

Only 35% of respondents knew that donor breastmilk had to be processed to make it safe for consumption by recipient babies. Knowledge on breastmilk banks and processing of DHM was significantly associated with health workers’ profession (Table 3). The practice of wet-nursing was also well-understood among the interviewed health workers. More than two-thirds (67%) of respondents had an adequate knowledge about wet nursing, and knowledge on wet nursing was associated with having a child (*p* = 0.023).

**Table 3:** Knowledge of breastmilk banks and infant feeding among health workers in Zimbabwean tertiary hospitals (*n* = 535)

	HEALTH WORKERS with adequate knowledge on wet nursing		HEALTH WORKERS who knew the concept of breastmilk banks		HEALTH WORKERS who knew about processing of donor breastmilk	
Profession	n	%	n	%	n	%
Nursing staff	177	72	182	76	79	32
Midwife	50	88	43	78	26	46
Nutrition staff	72	80	52	58	30	34
Doctors	9	47	15	83	14	74
General staff	44	39	71	66	34	30
Total	352	67	363	71	183	35
*p* value	*P* < 0.001		*P* = 0.005		*P* = 0.015	

All but one key informant had previously heard about human milk banks. Nursing managers and dieticians gave the correct scientific definition of breastmilk banks while other hospital managers, although correct, demonstrated layman’s ideas. In cases where knowledge on human milk banking was poor, donor breastmilk banks were confused with breastmilk expressed by mothers to feed their babies when they are away. One nursing manager articulated a breastmilk bank as:

*“A recognized service which is responsible for collecting, screening, processing and dispensing of human milk through prescription. The breast milk is donated by nursing mothers who are not the biological mother of the recipient infant.”* Nursing manager.

### Acceptance of donor human milk banking

The concept of DHM banking was fairly acceptable to health workers. About one-third (31%) reported that they would accept donor breastmilk to feed their own children, 47% would agree to donate or allow their partners to donate breastmilk through DHM banks, and 56% would encourage their clients to donate breastmilk to a DHM bank. Acceptance of donor human milk was associated with the participants’ health profession (*p* = 0.001) and level of knowledge on breastmilk banks (*p* = 0.009), but not associated with religion (*p* = 0.498), marital status (*p* = 0.714) or gender (*p* = 0.000). (Table 4).

**Table 4:** Acceptance of donor human milk banking among health workers in Zimbabwean Tertiary Hospitals (*n* = 527)

	HEALTH WORKERS willing to donate to a BM Bank		HEALTH WORKERS willing to accept DHM		HEALTH WORKERS willing to encourage mothers to use DHM	
Profession	n	%	n	%	n	%
Nursing staff	96	39	59	24	119	48
Midwives	33	58	18	31	35	62
Nutrition staff	53	59	24	27	61	68
Doctors	13	70	12	63	15	78
General staff	50	44	46	40	63	55
All Health Workers	248	47	163	31	295	56

Doctors demonstrated high acceptance of donor breastmilk banking (63%) compared to other professions (*p* = 0.001). Most health workers in the nutrition (59%), midwifery (58%), and medical fields (79%) reported a willingness to donate human breastmilk compared to health workers in the nursing (39%) and general fields (44%). Doctors (78%), midwives (62%) and nutrition (68%) professionals were more likely to encourage mothers to use donor human milk. Health workers with knowledge on the concept of human milk banks were more receptive to donate (*p* = 0.025), accept (0.009) and encourage (0.001) mothers to use donor breastmilk. More than half (56%) of the health workers reported they would encourage their clients to donate breastmilk to a DHM bank, although only 47% were willing to donate to a DHM bank themselves. Only 31% were willing to accept DHM for their own children.

All hospital managers (100%) accepted the concept of DHM banking. They reported that it was necessary to establish breastmilk banks in referral hospitals of Zimbabwe.

*“We need breastmilk banks to cater for babies whose mothers die within the first to second year of life and the remaining guardian cannot afford formula milk, and also for babies whose mothers cannot feed for medical reasons or are unable to produce adequate milk supplies”* Nursing manager.

Hospital managers also reported that donor breastmilk should be provided and made available as medical therapy, not for convenience.

*“Breastmilk banks are required in order to give newborn infants the best start in life and to achieve high breastfeeding rates. We should have a system that ensures availability of breastmilk as a form of medical therapy and not a convenience. We should treat breastmilk as a medicine which all children have the right to receive from birth”* Dietician**.**

### Reasons for health worker willingness or reluctance to donate or receive breastmilk from a breastmilk bank

Among the health workers who reported that they would donate breastmilk to a donor human milk bank, a common reason was to help other babies in need of breastmilk (80%). Some (41%) would not donate breastmilk due to fear of transmitting diseases to other children especially HIV. Table 5 shows common reasons why health workers would be willing to donate breastmilk to a human milk bank. Among health workers who would accept donor breastmilk, the majority (88%) would accept DHM because they regarded breastmilk as important for health and growth of children. Among those who rejected DHM, most health workers (61%) did so due to fear of transmission of diseases such as HIV (Table 6).

**Table 5:** Factors that influence health workers’ willingness to donate breastmilk to a donor human milk bank (*n* = 535)

*Reasons HEALTH WORKERS would agree to donate breastmilk to a bank*	n	%
Help other babies	193	80
Breastmilk is important	39	16
I have adequate knowledge on DHM banks	9	4
*Reasons HEALTH WORKERS would not agree to donate breastmilk to a bank*		
Fear of Diseases	121	41
Unacceptable	87	30
I have Inadequate knowledge	63	21
The practice is unethical	8	2
Decision lies with my partner	10	1
The practice is not safe	5	1

**Table 6:** Factors that influence health workers’ decision to receive DHM from a breastmilk bank (*n* = 518)

*Reason HEALTH WORKERS would accept to receive DHM*	n	%
Breastmilk is important to children	146	88
The practice is safe	19	11
I would accept after receiving more information	1	1
*Reasons HEALTH WORKERS would not accept to receive donor human milk*		
Fear of diseases	215	61
Unacceptable	80	23
Inadequate knowledge on DHM banks	30	9
Prefer formula milk	18	5
DHM not safe	9	3

### Perceptions on donor human milk banking

From the survey, more than half the health workers (57%) reported that breastmilk banks were important in Zimbabwe and half (50%) reported that the country could afford to introduce donor human milk banks (Table 7). There was general agreement that breastmilk banks were beneficial and most health workers (66%) reported that DHM banks reduced mortality and morbidity among preterm and orphan babies in hospitals (Table 7).

**Table 7:** Perceptions on donor human milk banking among health workers from tertiary Hospitals in Zimbabwe

Health Workers who responded yes to the statement;	n	%
Reduce morbidity and mortality of pre-terms and orphans in hospitals	349	66
BM banks are important in our hospitals	304	57
BM banks are affordable to set up in Zimbabwe	266	50
Breast milk donors should receive financial compensation when they donate breast milk	254	48
BM banks have safe breastmilk	230	44
Breast milk donors should receive non-financial compensation when they donate breast milk	223	42
BM banks are effective in removing all risk of disease or infection including HIV	181	35

Generally, health workers showed positive perceptions towards breastmilk banks, even though less than half (44%) indicated that DHM banks were safe. However, from interviews, hospital managers and dieticians had confidence in the safety of donor breastmilk.

*“Of course, donor human milk is safe* …*because it will be treated, and all possible harmful agents removed just like cow’s milk is processed when formula is being made”* Dietitian.

There was agreement among hospital managers that breastmilk donors were adequately screened, and donor human milk was treated and rendered safe to feed to infants. Appropriate storage conditions and measurements were reported as important factors to ensure the safety of donor human milk.

*“It can only be safe if there is proper screening for infections such as HIV and if proper storage is ensured”* Clinical Director.

### Perceptions of health workers on donor human milk banking in a high HIV setting

**Fig. 2:**
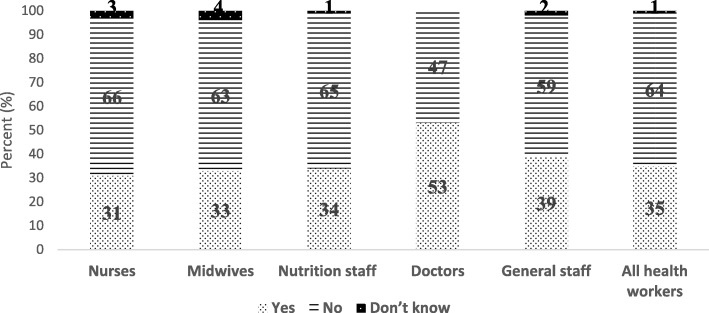
Perceptions of health workers on whether treatment and processing donor human milk could eliminate HIV

Table 7 shows health workers’ views on elimination of HIV during donor human milk processing. Only 35% of health workers reported that HIV could be eliminated during treatment and processing of DHM. More than half of the doctors (53%) were aware that HIV and other diseases were eliminated during processing of DHM. Perception on elimination of HIV in DHM during processing was associated with health profession (*p* = 0.036) but not associated with the level of knowledge of breastmilk banks (*p* = 0.167). Most respondents from other professions among health workers other than doctors were not aware that treatment of donor breastmilk eliminated HIV (Fig. 2).

Most of the hospital managers reported that donor breastmilk could be made safe and free from HIV if donors were screened and donor breastmilk underwent proper treatment processes and storage. Other hospitals managers perceived donor human milk banking as a cost-effective strategy and a more beneficial means to feed infants born to HIV-infected mothers.

*“Donor breastmilk is actually a cost-effective means of ensuring children born to HIV positive mothers receive breastmilk. As opposed to sourcing infant formula as an alternative feed which is costly and requires certain conditions for preparation and storage, increasing risk of contamination and malnutrition”* Dietetics and nutrition department manager.

There was consensus among hospital managers that Zimbabwe was in a position to treat and process donor human milk in the referral hospitals if resources were provided and priorities set by the government and the private sector.

One clinical director and a nursing manager reported:

*“It is possible to safely treat and process donor breastmilk even in this setting where HIV rates are high if resources are available, both human and material and if priorities are made up correctly”.*

### Factors influencing acceptance of donor human milk banking among health workers

Health workers’ willingness to encourage mothers to use donor human milk was positively associated with increased knowledge on DHM banking (*p* = 0.002) and prior exposure to a worksite with a DHM bank (*P* = 0.015). Health workers with knowledge on DHM banking and those who had previously worked in a setting where a breastmilk bank was present were 52% (OR 1.52 CI 1.34; 1.78. *p* = 0.002) and 36% (OR 1.36 CI 1.16; 2.82. *p* = 0.015) more likely to encourage mothers to use donor breastmilk respectively. Religion (*p* = 0.624) and awareness of DHM banking (*p* = 0.52) did not influence health workers’ acceptance to DHM.

### Readiness and commitment of tertiary hospitals to implement donor human milk banking

Referral hospital management staff for Parirenyatwa and Harare Central Hospitals were committed and eager to have breastmilk banks established to improve breastfeeding rates and provide a chance of feeding breastmilk to infants unable to be breastfed for medical or other reasons. Hospital managers reported that the commitment to establishing breastmilk banks was high.

*“Challenges like unavailability of human and material resources, equipment, limited or improper infrastructure need to be addressed to make it possible*” Clinical director and nursing manager.

One clinical director highlighted that hospital management were aware of the need to establish breastmilk banks as the issue had been raised during planning meetings the year prior to the current study. Other middle-level managers reported that although commitment towards breastfeeding initiatives among hospital decision makers was often reported as high, actual efforts to provide resources was very low. This comment reflected the limited support given to policies that promoted breastfeeding such as the BFHI. Another key informant at middle management level reported that referral hospitals’ commitment to breastfeeding or even establishing a breastmilk bank seemed low because of the lack of resources and lack of knowledge on the importance of breastfeeding among health workers.

*“A lot of education is needed for staff members before this can be done. Too many misconceptions about the milk banks”* Dietician.

Respondents felt that the government and nutrition community in the country needed to act swiftly to move forward the agenda on donor human milk banking. One clinical director stated that, *“the issue of breastmilk banks is now long overdue for a country and a city like Harare with so many infants and neonates who may benefit from it.*”

## Discussion

This study showed that health workers were more receptive to donating DHM and more likely to encourage others to use DHM banks than receiving DHM themselves. Only about one third (31%) of health workers reported they would accept donor breastmilk to feed their own children, while 47% would agree to donate or allow their partners to donate breastmilk to DHM banks. Fifty-six per cent indicated they would encourage their clients to use DHM banks. Amongst the professions interviewed, doctors demonstrated the highest acceptance of DHM banks. Knowledge on DHM banks and prior work at a health facility with a DHM bank was shown to positively influence acceptance of DHM banking. Helping babies in need of breastmilk was identified as the major reason to donate breastmilk, while the fear of HIV transmission was the major barrier to DHM banking acceptance.

Most health workers were either aware of, or had adequate knowledge of DHM banking, based on their personal knowledge and work experience. Professional knowledge and experience with blood banks may have contributed to their knowledge on DHM banks. This finding indicated limited knowledge on infant feeding options among tertiary-level health workers which is detrimental to national progress in child health. In a country where breastfeeding is highly regarded and the BFHI program is being advanced, there was need for extensive investment on infant feeding education among health professionals [40]. Considering most mothers relied on health workers for information on DHM and breastmilk feeding, it was important for health workers to have adequate knowledge on the subject [6, 32, 34]. Considering many working mothers in urban resource-limited settings needed strong encouragement to use expressed breast milk, such an investment in health professional education should be essential [4, 41, 42]. Education on Infant and Young Child Feeding, and components on use of DHM for medical purposes could be incorporated during in-service training for health professionals.

Findings from interviews with hospital management and key opinion leaders in the health sector demonstrated high acceptance of DHM banks. This was important considering most resource-limited countries were considering health interventions that save lives and reduce health costs, both of which are commonly associated with DHM banks. Brazil’s comprehensive DHM bank approach saves the country about US$540 million annually in medical costs [4, 43, 44]. Among the main referral hospital staff in Zimbabwe, acceptance of DHM banking was high among doctors and fair among other health professionals. Doctors also demonstrated increased knowledge of DHM and adequate knowledge on processing of DHM compared to other health professionals. Health workers with increased knowledge on the concept of DHM banking were more receptive to DHM banks and indicated the need for a greater level of knowledge as critical to motivate acceptance of DHM banking [32].

The main barrier to DHM banking was the fear of infectious diseases. This finding was consistent with studies elsewhere where safety of DHM was a major obstacle to DHM banking [6, 45]. A few health workers believed that donor breastmilk was safe and its treatment could eliminate disease pathogens including HIV. Most health workers were more comfortable with encouraging clients to use DHM banks or donating breastmilk themselves than accepting DHM for their own children. This finding reflected a fear of disease and lack of familiarity with processes involved in DHM treatment which could be addressed through health education programs.

A few health workers reported that the practice of DHM banking was unacceptable. Such beliefs could potentially affect general acceptance of DHM banks by communities. To ensure success of DHM banks in Zimbabwe, education on the principles of the banks is a pre-requisite to ensure health workers’ trust in the practice. Opinions of mothers and caregivers on DHM are mainly influenced by health workers as indicated elsewhere [5, 34, 42, 46]. Hence getting health workers on board is the entry point for DHM banks in settings where HIV prevalence is high and strong cultural perceptions on infant-feeding exist. Lack of familiarity with DHM banking was a major obstacle to acceptance of DHM by mothers in a similar setting in South Africa [6].

Even with some misconceptions on safety, the fact that most health workers were keen to encourage mothers to use DHM reflected the potential success of the DHM bank initiative. This encouragement was despite the fact that more than half of respondents believed that DHM was not safe and could not be processed to eliminate HIV and other pathogens (Table 7). Doctors and midwives were more receptive to DHM banking possibly due to extended period of education and experience in the clinical setting [47, 48]. It requires seven to nine years to qualify as a medical doctor or midwife in Zimbabwe, during which time exposure to infants with special needs in NICUs is high. Contrary to prior assumptions, staff in the nutrition field were not highly knowledgeable and accepting of DHM banking, possibly because other nutrition support staff such as hospital food service supervisors were less exposed to breastfeeding and NICUs.

Fear of HIV is potentially the main barrier to accessing DHM banks, and this was anticipated as Zimbabwe is a high HIV setting [3, 6, 31]. Information from a clinical director at one of the tertiary institutions revealed that the country had formerly established DHM banks in the 1990s which were closed during the initial stages of the HIV epidemic [16]. Since then, technological advances and the pasteurization of DHM can eliminate HIV in EBM. Consequently, hospital managers and health sector key opinion leaders did not view HIV as an obstacle to DHM banks as it can be eliminated during treatment and donors are rigorously screened [49–51]. The current requirement is government's commitment and investment in DHM treatment and processing to ensure success of the initiative.

Comments by managers that commitment to investment in breastfeeding by the government was insufficient and that there was a lack of knowledge on infant feeding among health workers highlights the importance of education around protecting, promoting and supporting breastfeeding. An integrated DHM bank model with newborn care services could improve the country’s breastfeeding rates, reduce the healthcare cost of managing preterm babies, and improve child health care [29, 52]. Hygiene and handling of DHM by caregivers needs critical attention considering that water and sanitation infrastructure have deteriorated in such settings [53]. Other authors have argued that DHM could be expensive when compared with formula milk [52], and that feeding DHM collected from full term mothers late in lactation could result in milk composition that is unsuited to the needs of preterm infants and requires additional human milk fortifiers [54]. Despite such challenges, DHM particularly exclusive-human milk diets, offer infants better protection from the risk of necrotizing enterocolitis, sepsis and feeding intolerances [1–3, 23, 26].

Participants recruited for this study were limited to health workers in tertiary hospitals and potential sites of DHM banks. Consequently, the sample was not representative of health workers in the country. Other groups with direct influence on mothers’ infant feeding decisions such as husbands and mothers-in-law were not included in the study.

## Conclusions

Donor human milk banks were acceptable to health workers, and the level of acceptance was higher among doctors, health workers with adequate knowledge of DHM banking and health sector policy reformers. While a willingness to establish DHM banks in major referral hospitals was high, limited finances and facilities and a fear of HIV infection were potential barriers. Consequently, financial commitment from authorities, and education among health workers on the safety of DHM is crucial to the success of DHM banking in HIV endemic areas.

## Data Availability

The datasets used and analysed during the current study are available from the corresponding author upon reasonable request.

## Abbreviations

BFHI: Baby Friendly Hospital Initiative
BM: Breastmilk
DHM: Donor human milk
EBF: Exclusive Breast Feeding
HIV: Human Immune Deficiency Virus
NICU: Neonatal Intensive Care Units
WHO: World Health Organisation
